# Supplementary material for: Genetic comparisons of interleukin-17 reveal a framework for complex signaling evolution
Source: bioRxiv. 2026 Apr 14:2026.04.13.718218. Preprint. [Version 1] doi: 10.64898/2026.04.13.718218 (PMC13105070; doi:10.64898/2026.04.13.718218)
Supplement: Supplement 3 [file media-3.zip › Supplemental files for paper/ERC/IL17xIL17_comparisons/IL17RD_IL17RE.pdf]

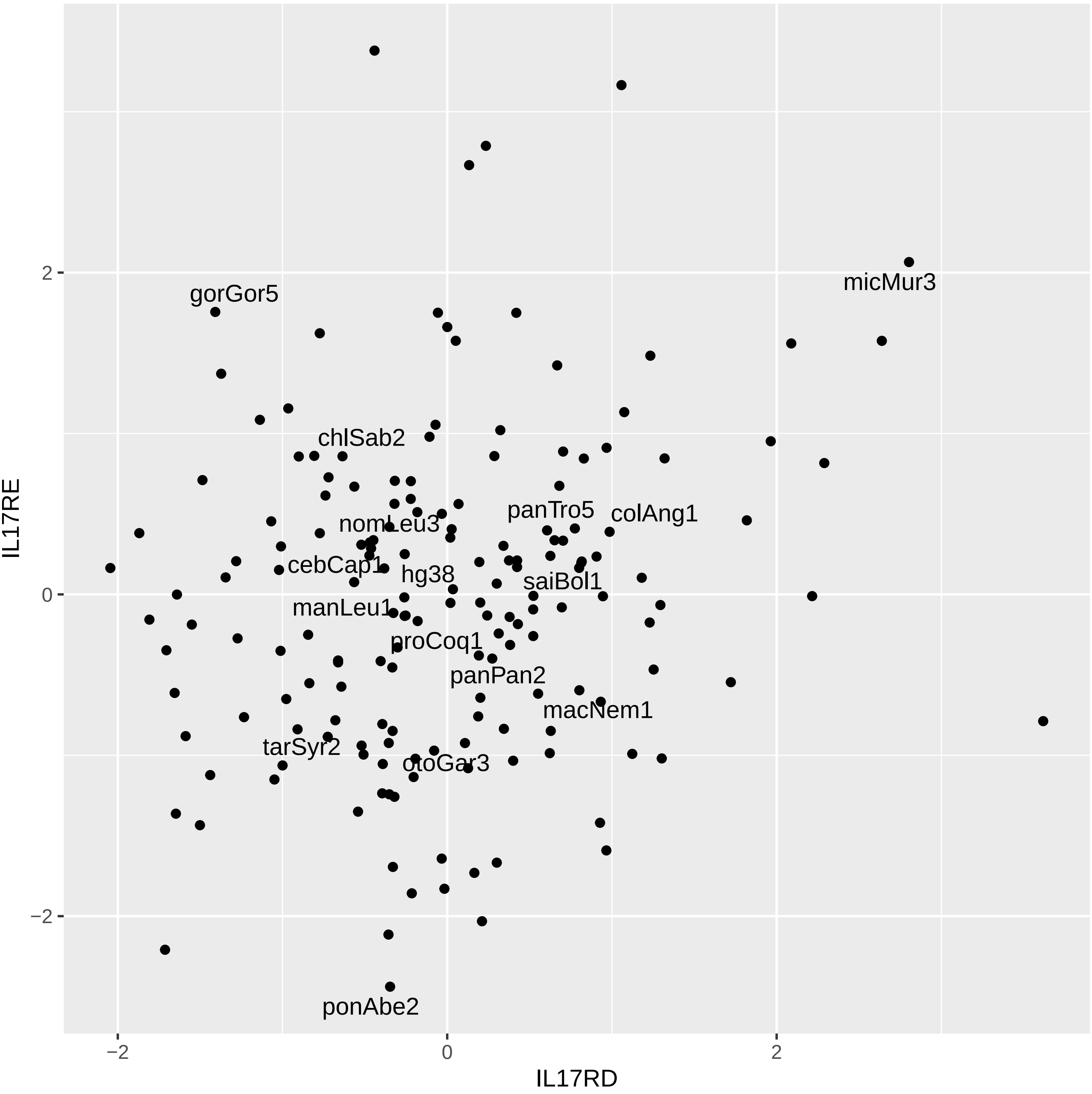

IL17RE
IL17RD
2
0
−2
−2
0
2
gorGor5
micMur3
chlSab2
panTro5
colAng1
nomLeu3
cebCap1
hg38
saiBol1
manLeu1
proCoq1
panPan2
macNem1
tarSyr2
otoGar3
ponAbe2
